# If there’s a penis, it’s most likely a man: Investigating the social construction of gender using eye tracking

**DOI:** 10.1371/journal.pone.0193616

**Published:** 2018-03-01

**Authors:** Frederike Wenzlaff, Peer Briken, Arne Dekker

**Affiliations:** Institute for Sex Research and Forensic Psychiatry, University Medical Center Hamburg-Eppendorf, Hamburg, Germany; University of Lethbridge, CANADA

## Abstract

In their foundational work on the social construction of gender, Kessler and McKenna (1978) investigated the relationship between gender attribution and genital attribution. We used digital reproductions of the original stimuli to replicate their findings in the current social context. To further investigate the underlying decision processes we applied eye tracking. The stimuli shown varied in the composition of gender cues: from those more commonly associated with maleness to associated with femaleness. Applying the ethnomethodological approach originally used, participants were asked to decide for each stimulus whether they saw a man or a woman and to indicate subjective confidence with the decision. In line with the original results we found that the genital attribution contributed immensely to the gender attribution. Also, male gender was ascribed more often when the penis was present than was female gender when the vulva was shown. Eye tracking revealed that overall most dwell time as a proxy for important information was dedicated to the head, chest and genital areas of all the stimuli. Total dwell time depended on whether the gender attribution was made in line with the depicted genital, if the genital was a penis. Attributing female gender when a penis was present was associated with longer total dwell time, unlike attributing male gender with a vulva shown. This is indicative of higher cognitive effort and more difficulty ignoring the penis as opposed to the vulva. We interpret this finding in context of the persistent male dominance as well as to the socio-cultural understanding of the vulva as a concealed and therefore seemingly absent organ. In summary, we were able to show that the gender attribution is still closely linked to genital attribution when having a binary forced choice task and that the penis is a special cue in this attribution process.

“Gender attribution is, for the most part, genital attribution; and genital attribution is essentially penis attribution“–Kessler and McKenna, 1978

## Introduction

Every time we interact with someone or even just see them from a distance, we always attribute gender–that is, we decide whether a person is male or female [1]. Indeed, the discrimination of gender is considered one of few automatic and habitual aspects of person perception [2]. Although scholars have acknowledged the dimensional structure of many gender differences [3–5], people tend to categorize men and women on the basis of sex to simplify a complex world [6]. Because we do not usually see or know about each other’s genitalia, the attribution actually depends on indirect cues of the anatomical genitals as observed in facial structure, voice, dress, assumedly gendered behavior or social context. This not directly visible “cultural genital” [7] which is expected to be there “exists in a cultural sense if the person […] is assumed to have it.” ([8], p. 154).

The concept of this social construction of gender, a then radical idea, was introduced into academic debate in the 1970s, when social inequality was occasionally backed up by biological differences between men and women [9, 10]. During that time the social psychologists Suzanne J. Kessler and Wendy McKenna built up-on Garfinkel’s work and published their foundational book *Gender*: *An Ethnomethodological Approach* [8]. To unravel the relationship between gender attribution and genital attribution and to possibly collect additional information about the relative importance of physical characteristics in deciding gender, they designed their so-called *overlay study* (p. 145).

Although their work has been very influential and remains important until today [11–13], their results have never been replicated. Hence, the first aim of our study was to investigate its empirical validity several decades later. By placing 11 different plastic overlays on top of each other, Kessler and McKenna created a total of 96 figures with varying amounts gender cues commonly associated with maleness or femaleness. These ranged from “exclusively male” (short hair, flat chest, narrow hips, body hair and penis) to “exclusively female” (long hair, breasts, wide hips, little body hair and vulva; Kessler and McKenna used the term vagina, but we decided to use the scientifically correct term for the whole exterior genitalia, which is vulva). For each figure participants were asked “Is this a picture of a female or a male?”. Not only were the numerical amounts cues important for participants’ decisions, but also–and more essentially–the relative impact of the cues was important. Based on their results, Kessler and McKenna stressed the outstanding importance of the penis in the decision-making process, since a male gender attribution was almost always made when a penis was present. Nevertheless, it is social practices, not body parts, that constitute the foundation of the gender attribution, which in itself is ongoing, suggesting that its accomplishment is “always practical, in flux, and situated” ([1], p.180). Such practices are interwoven with commonsense understandings or “natural attitudes” [14]. In the 1970s those “natural attitudes” about gender, which were assumed to be objective facts, included that there are two (and only two) genders and that genitals are the essential sign of gender ([7], pp. 122–128).

These assumptions have been challenged and intensively debated in the past three decades. Especially since non-binary characters now appear in mainstream media, awareness about the existence of more than two genders is no longer exclusive to academic fields [15]. Still, the question of what constitutes gender, how/if it is different from sex and even how many sexes/genders there are remains subject to debate (e.g. [16–19]), at times leading to conceptual muddles [20]. Especially from a social constructionists’ point of view, the complexity of sex and gender or sex/gender becomes obvious [11]. Additionally, different social developments have led to a strong increase in gender equality [9, 21, 22] over the past years in many western countries. We propose those social changes might have led to a change in the “natural attitudes”. Hence, we would expect that the penis is still important for gender attribution, but not as important as it used to be.

Since the 1970s technology has evolved, offering the possibility to directly track eye movements during decision processes [23]. We have applied eye tracking in the replication of Kessler and McKenna’s study. In eye tracking research the location of the central fixation is considered a good proxy for the focus of attention [24, 25]. It is also well established that more fixations and fixations of longer durations are directed to regions that are more informative for the task, resulting in longer total dwell times [26, 27]. Entry time or time to first fixation as well as the duration of this first fixation is usually considered as automatic and not consciously controllable. Faster entries to the relevant regions and longer first fixations are indicative of novelty or greater interest [23]. Eye tracking has only recently been introduced to the field of sex research but was recommended for the investigation of socio-cultural questions [28]. The only three studies measuring eye movement during gender attribution for whole body images instead of just single, isolated cues have presented schematic stimuli without faces or visible genitals [29–31]. In the absence of those cues, body shapes, especially the waist-hip-ratio (WHR) were used to infer gender. This ability to categorize gender based on WHR apparently develops as early as between the ages of 4 and 6 [30]. If the WHR was not available, the shoulders and the pelvis region were looked at to infer gender of bodies in motion [29]. The WHR has also been investigated in eye tracking studies with regard to sexual attractiveness ratings. Men have for example been found to fixate more and longer on images of clothed women with a lower WHR and to select them as more attractive [32]. With nude images, men attended more to the midriff and the buttocks for back-posed images of women and to the breasts for front-posed images [33]. When presented with pictures of models in casual clothing but no additional task, participants generally tend to look at faces first [34–36]. However, when nude models are presented some allocation of attention is shifted to the body, away from the faces [37, 38].

In the present study we are the first to measure eye movements during gender attribution for stimuli with faces as well as primary and secondary gender cues available. To date, the relative importance of the different gender cues, especially penis and vulva, during visual inspection in the decision process is unknown. Although the face is considered to be of special importance in gender attribution [39–41], we assume that attention shifts away from the face to the chest, WHR and genital area, as it has been demonstrated for other tasks with explicit stimuli [37, 38].

Given the scientific and social progress of the past decades, the main interest of the present study is whether Kessler and McKenna’s results hold almost 35 years later. We consequently employ their study design to (1) compare their results to those obtained in the current social context and (2) use eye tracking technology to gain insights into the viewing behavior during the process of gender attribution. Replicating the original design also meant that we used the binary forced choice task and referred to “male” and “female” as “the other gender”, despite acknowledging that there are more than just these two genders.

We hypothesized that in such design (1) gender attribution is still largely genital attribution and that the penis remains important. Concerning eye movements during attribution processes, we (2) expect most dwell time to be directed at the areas most relevant for gender attribution, namely the head, chest, waist-hip-region and genitals. Also, if gender attribution is indeed predominately genital attribution, we (3) await differences in viewing behavior on stimuli with a penis compared to those with a vulva. Lastly, we were interested if (4) viewing patterns differed between participants who attributed gender in line with the depicted genital and those who did not.

## Materials and methods

### Participants

A total of 40 participants (50% female) ranging in age between 20 and 64 years (*M* = 30.15, *SD* = 9.64) were tested. All participants self-identified as exclusively or mostly heterosexual, and as exclusively heterosexual according to behavior and fantasy [42]. Participants were recruited via professional and personal contacts. No compensation was given. A statistical power analysis was performed for sample size estimation. Based on data from the original overlay study, we assumed large effects using Cohen's criteria [43]. With an alpha = .05 and power = 0.80, the projected sample size needed with this effect size is approximately N = 38. Thus, our sample size was adequate for the main objective of this study.

### Stimuli and apparatus

#### Stimulus material

**Fig 1:**
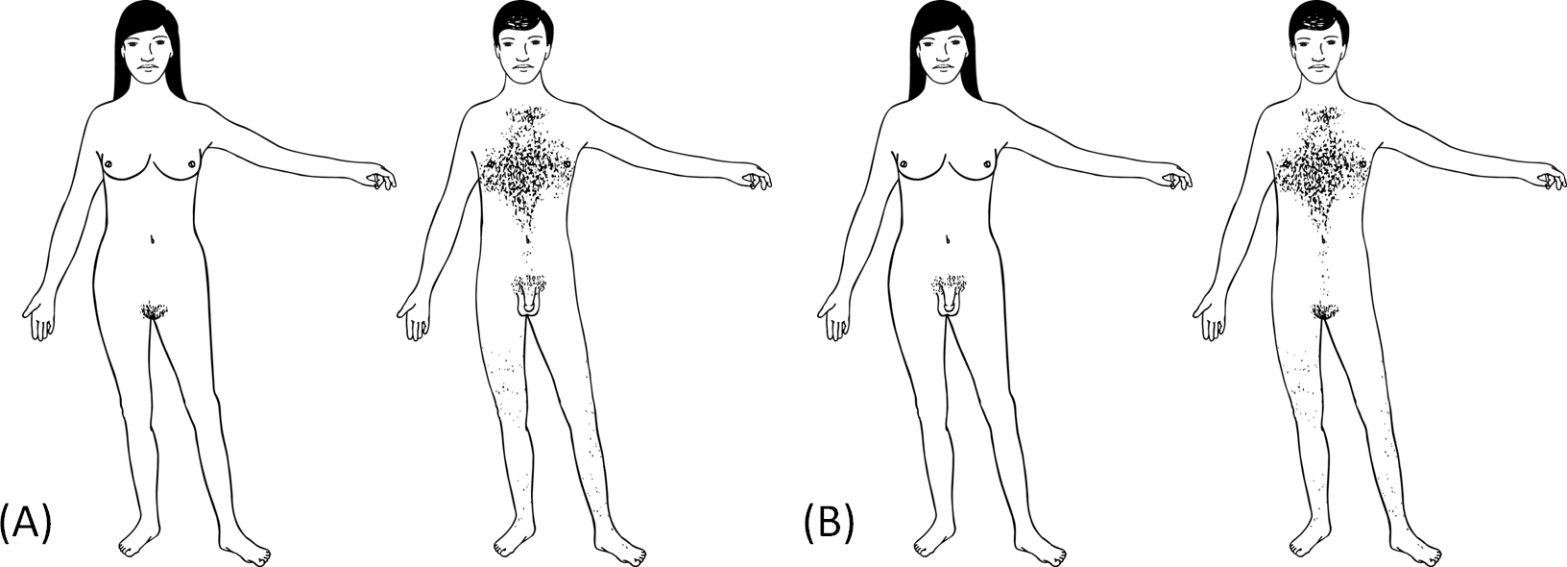
Stimuli in the congruent condition (A) and the incongruent condition (B).

We took the two example stimuli published in the original overlay study [8] to build a digital replication of some of the described stimuli, using Adobe Illustrator CS6. While all the stimuli had the same, non-gender-specific face they differed with regard to the combinations of gender cues. The five variable cues were hair (long hair vs. short hair), hips (wide vs. narrow), chest (breasts vs. flat chest), body hair (no body hair vs. body hair) and genital (vulva vs. penis). Some stimuli had a neutral t-shirt or pants. In this text, the stimuli containing exclusively characteristics that are more commonly associated with maleness or femaleness are referred to as the congruent stimuli. The final stimulus set consisted of 32 black-and-white schematic drawings of adults with varying gender cues within an area of 300 × 420 pixels presented against a white background. For the purpose of this study only the two supposedly congruent stimuli and their incongruent counterparts displaying the other genital (penis instead of vulva and vice versa) are analyzed (see Fig 1).

#### Eye tracking

Stimulus presentation and data collection were conducted on a 24-inch widescreen computer monitor (Dell P2213, 32 BIT true color, refresh rate 59 Hz) with a resolution of 1680 × 1050 pixels, using SMI (SensoMotoric Instruments GmbH, Berlin, Germany) software ExperimentCenter 2^TM^.

An eye-tracker collected the eye data using corneal reflection and dark pupil with a video-based infrared eye camera at a sampling rate of 120 Hz with a spatial accuracy of approximately 0.5°. The SMI RED system (SensoMotoric Instruments GmbH, Berlin, Germany) is a contact-free, remote-controlled eye tracking device with automatic eye and head tracker to automatically compensate for slight head movements. No headrest was used, and viewing distance varied according to the most comfortable position for the participants. Eye movements were only recorded when looking at the stimuli and not while performing other tasks. The iView X default settings were used.

### Procedure

Tested individually by a trained experimenter, participants were informed that they would see a series of images consisting of schematic drawings of nude adults with varying gender cues. The task was explained verbally before the measurement began to ensure understanding and was repeated on screen directly before the assessment. After giving socio-demographic information (age, gender) participants were asked to sit comfortably leaning back to minimize body movements. Following, a standard five-point calibration procedure was performed. Calibration was validated with the included software and repeated when necessary until the optimal calibration criterion was reached.

To familiarize participants with the procedure and to avoid eye movements due to novelty, one practice trial was conducted with a completely dressed figure with short hair. Subsequently, the congruent stimuli were randomly presented at positions 1 and 2, followed by the other 30 stimuli in randomized order.

For each stimulus participants were asked to press space as soon as they had decided whether the figure is more likely to be male or female. Participants had to indicate their decision by choosing one of the two gender options and then click a button to continue. After their self-paced reaction or a maximum time of 15 *seconds* a new screen appeared. On the next screen, they were asked to rate their subjective confidence with the decision on a 4-point scale ranging from 1 (*very confident*) to 4 (*very unconfident*). All answers were forced choice and no feedback of any kind was given.

After seeing all stimuli, participants were asked for a short written statement on how they proceeded when attributing gender. Finally, sexual orientation was assessed on three dimensions: orientation, behavior and fantasy. Participants were thanked for participation and dismissed. Depending on the participant’s speed, the whole experiment took between 10 and 25 minutes to complete.

### Ethics statement

All experimental procedures complied with the Declaration of Helsinki. Ethical approval was formally obtained from the ethics commission of the Psychotherapists Association Hamburg (Psychotherapeutenkammer Hamburg). All participants provided written informed consent prior to study participation.

### Data reduction and data analysis

#### Gender attribution and subjective confidence

To account for repeated measures taken from the same subject, gender attributions were subjected to a mixed logistic regression with fixed effects for the categorical variables participant gender (male vs. female), stimulus congruence (congruent vs. incongruent) and stimulus genital (vulva vs. penis) as well as all their two-way interactions and the three-way interaction.

Subjective confidence was examined with ordinal logistic regression using the fixed effects of stimulus congruence (congruent vs. incongruent), stimulus genital (vulva vs. penis) and their interaction. The incongruent stimulus with the penis was used as the reference category.

#### Eye tracking

Visual attention was examined with the software packages SMI BeGaze 3.2.28. The standard settings of a minimum duration of 80 milliseconds and a maximal dispersion of 100 pixels were used to define fixations. As no fixation cross was used, first fixations were excluded via BeGaze prior to data analysis. This was done to ensure that the first fixation analyzed was not simply an error due to carryover of the eye position after completing the previous task and before the stimulus onset.

**Fig 2:**
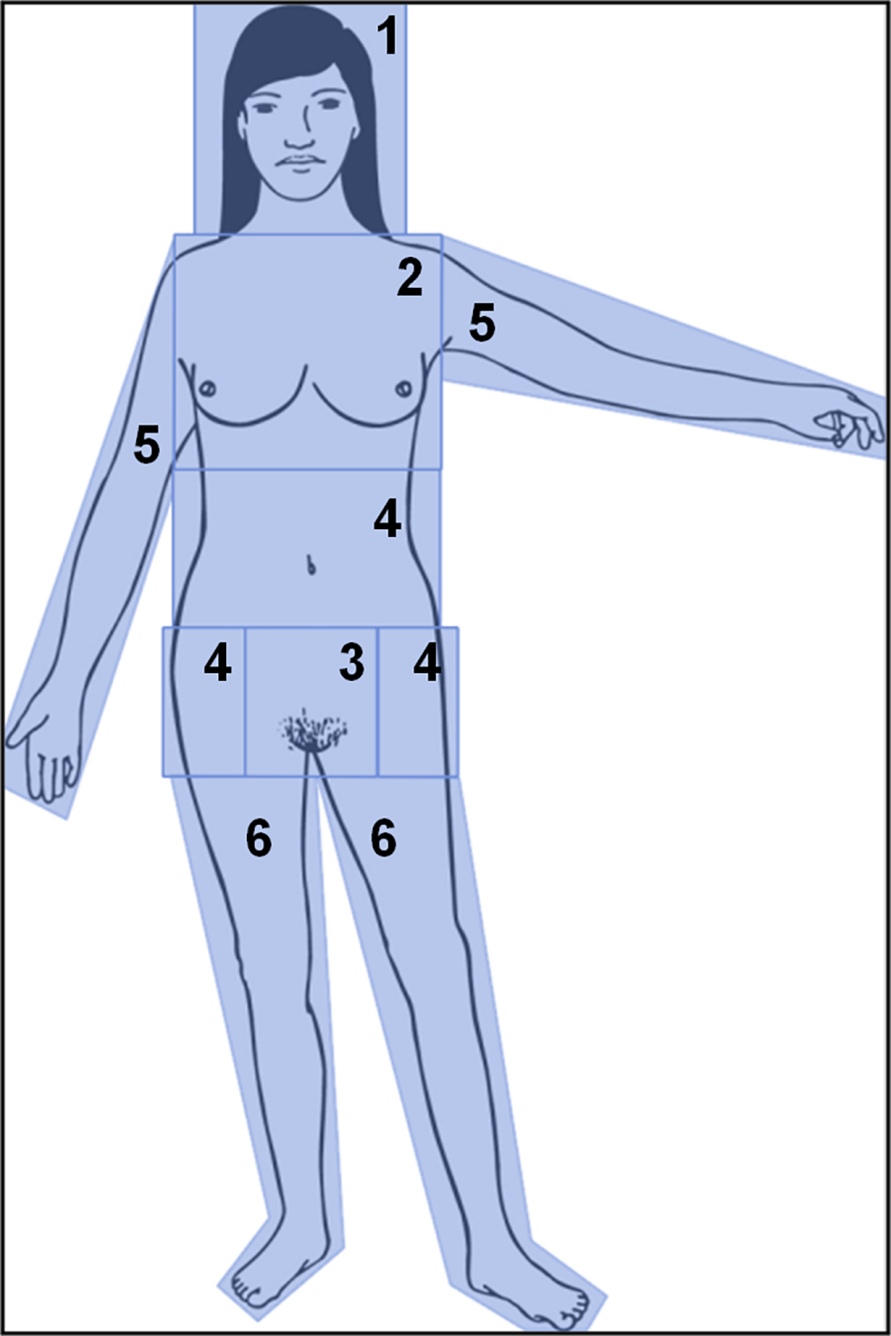
Applied areas of interest. Areas of interest were assigned to (1) head, (2) chest, (3) genital, (4) waist, (5) hips, (6) arms, (7) legs.

For subsequent analysis of the eye tracking data the stimulus image was divided into seven areas of interest (AOI) according to the variations in gender cue: (1) head, (2) chest, (3) genital, (4) waist, (5) hips, (6) arms, and (7) legs (Fig 2). In each of the regions, the relative amount of time spent viewing the area (dwell time in *ms*) was measured. To allow for direct comparison, the same AOI were used for all stimuli. All trials with less than 50% dwell time directed at these AOI (10%) as well as all reaction times two *SD* slower than the mean (4%) were excluded.

Relative dwell time averaged over all four stimuli was highest for the head (26%), followed by the chest (21%) and the genital (18%). For the waist-hip-region, arms or legs, relative dwell time did not exceed 3%. Hence, only the three areas of head, chest and genital are considered for subsequent analysis.

The dependent eye movement measures of total dwell time, relative dwell time, entry time, number of fixations, first fixation duration and average fixation duration were each subjected to a generalized linear mixed model analysis (GLMM; GENLINMIXED in SPSS) to account for repeated measures of the same subject. Fixed effects of the categorical variables stimulus congruence (congruent vs. incongruent), stimulus genital (vulva vs. penis), AOI (head vs. chest vs. genital), all of their two-way interactions and their three-way interaction were considered.

Since almost all attributions made for the congruent stimuli were in accordance with the depicted genital, only the incongruent stimuli were used to further analyze differences between the participants who attributed in agreement with the stimulus genital and those who did not. Single models were calculated for all dependent eye movement measures with fixed effects for stimulus genital (vulva vs. penis), AOI (head vs. chest vs. genital), attributions in line with the genital (in line vs. not in line), all of their two-way interactions and their three-way interaction.

We used backwards elimination to remove all non-significant factors from the models. Only the results of the final models are reported. A two-level alpha of 0.05 was used for all statistical tests. Odds ratios (OR) with 95% confidence intervals were calculated where suitable. For mixed model analyses, random intercepts were assumed for subjects allowing for individual differences between participants. Estimated marginal means with 95% confidence intervals were computed for the dependent variables followed by multiple pairwise t-tests to compare means. All analyses were performed using SPSS (Version 22, release 22.0.0.2, IBM Corp., USA).

## Results

First, for the gender attributions and the subjective confidence ratings, we tested whether we could replicate the general tendency concerning the dominance of the penis in the attribution process. Second, we analyzed different eye movement measures to further investigate the viewing patterns for the different stimuli.

### Gender attribution and subjective confidence

#### Gender attribution

While for the congruent stimuli almost all participants attributed gender in line with the depicted genital, this was not the case for the incongruent stimuli (see Table 1). However, the incongruent stimulus with a penis (and all other gender cues more commonly associated with femaleness) was rated as male by 75% of the participants, while only 55% rated the incongruent stimulus with a vulva (and all other gender cues male) as female. The mixed logistic regression analysis revealed significant effects of stimulus congruence and stimulus genital (see Table 2).

**Table 1:** Gender attribution in line with the depicted genital and subjective confidence.

Congruence	Genital	Attribution in line with the Genital	Subjective Confidence				
			Very Confident	Somewhat Confident	Somewhat Unconfident	Very Unconfident	
**Congruent**							
	Vulva	92.5%	82.5%	12.5%	5.0%	-	
	Penis	100%	97.5%	2.5%	-	-	
**Incongruent**							
	Vulva	55%	27.5%	37.5%	22.5%	7.5%	
	Penis	75%	17.5%	32.5%	42.5%	12.5%	

*Note*. n = 40.

**Table 2:** Influences on the gender attribution in line with the genital.

	OR	95% CI	p
**Stimulus Genital**^**a**^	3.36	[1.28, 8.85]	.014
**Stimulus Congruence**^**b**^	0.05	[0.01, 0.20]	< .001

*Note*. OR = odds ratio. CI = confidence interval.

^a^ penis vs. vulva

^b^ congruent vs. incongruent

Odds ratios indicate that the change in the odds of attributing in line with the depicted genital is 0.05 for stimulus congruence (see Table 2). In other words, responses in accordance with the stimulus genital were 20 times less likely for the incongruent compared to the congruent stimuli. The odds of responding in line with the depicted genital were 3.6 times more likely if a penis was present compared to if a vulva was present.

Neither participant gender nor age had a significant effect on the gender attribution and were therefore not included in subsequent analyses.

#### Subjective confidence

Concerning participant confidence in their gender attributions, most participants (67.3%) stated they were “very confident” for congruent stimuli, while fewer (18%) responded that way for the incongruent condition (Table 1).

The ordinal logistic regression revealed that subjective confidence depended on stimulus congruence and stimulus genital, as their interaction predicted a higher confidence rating (see Table 3). We found higher odds for higher confidence ratings for congruent stimuli to be precise, 172 times higher for the stimulus with a penis and 20 times higher for the stimulus with a vulva. The difference between the congruent stimuli was not significant (*p* = .052). Although the odds for higher confidence ratings were slightly higher for the incongruent stimulus with a vulva compared to the one with a penis this difference was also not significant.

**Table 3:** Influences on subjective confidence.

Congruence	Genital	OR	95% CI	p
**Congruent**				
	Vulva	20.54	[7.23, 58.36]	< .001
	Penis	172.01	[21.08, 1403.57]	< .001
**Incongruent**				
	Vulva	1.58	[0.71, 3.53]	.260
	Penis	1	-	-

*Note*: OR = odds ratio; CI = confidence interval.

### Eye tracking data

#### Eye tracking outcomes comparing all stimuli

Concerning the different eye movement measures we found effects for total dwell time. The two-way interaction of stimulus congruence and area of interest (AOI) was significant, indicating that time looking at the AOIs differed between the congruent and the incongruent stimuli. Descriptive results are shown in Table 4, and the corresponding model results are shown in Table 5.

**Table 4:** Dwell time [ms] by stimulus congruence and AOI.

Congruence	AOI	M (SE)	95% CI
**Congruent**			
	Head	449.67 (56.95)	[337.71, 561.63]
	Chest	262.94 (56.95)	[150.98, 374.90]
	Genital	210.27 (56.95)	[98.31, 322.23]
**Incongruent**			
	Head	401.83 (58.19)	[287.43, 516.22]
	Chest	503.45 (58.19)	[389.06, 617.85]
	Genital	429.15 (58.19)	[314.76, 543.54]

*Note*. AOI = Area of Interest. CI = confidence interval.

**Table 5:** Influences on dwell time.

	F	df	p
**All Stimuli**			
**Stimulus Congruence**^**a**^	13.74	1, 402	.000
**AOI**^**b**^	2.90	2, 402	.056
**Stimulus Congruence x AOI**	6.56	2, 402	.002
			
**Incongruent Stimuli**			
**Accordance of Attribution**^**c**^	5.43	1, 194	.021
**Stimulus Genital**^**d**^	2.38	1, 194	.125
**Attribution x Stimulus Genital**	7.33	1, 194	.007

^a^ congruent vs. incongruent

^b^ head vs. chest vs. genital

^c^ in line with depicted genital vs. not in line

^d^ penis vs. vulva

**Fig 3:**
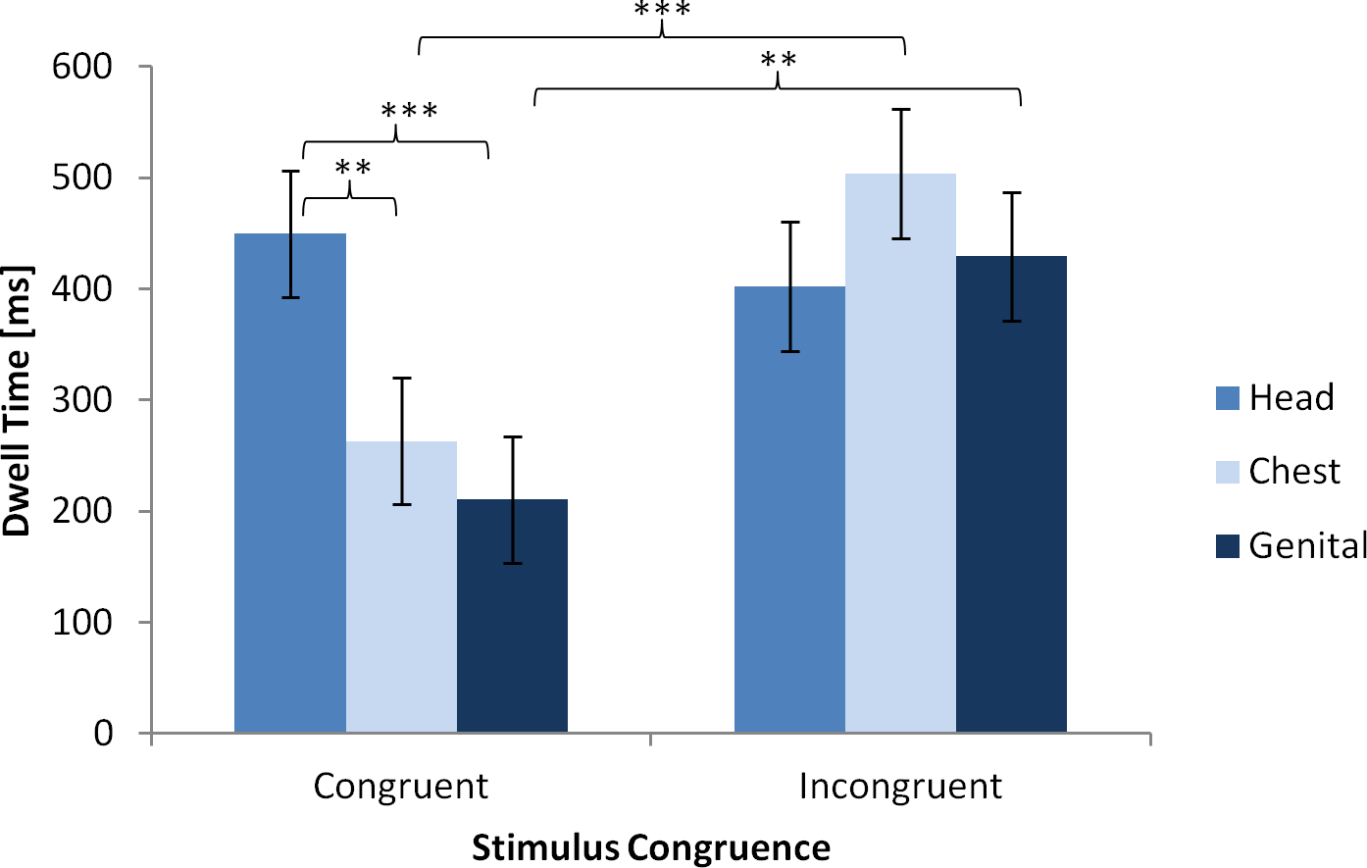
Means and SDs of dwell times [ms] as a function of stimulus congruence and area of interest [ms]. ** p < .01, *** p < .001.

Participants dwelled longer on the chest and genital of the incongruent stimuli than on those areas of the congruent stimuli (*t*(402) = 3.81, *p* < .001 and *t*(402) = 3.46, *p* = .001, respectively; see Fig 3). Also, with the congruent stimuli, participants spent more time looking at the head than on the chest or the genitals (*t*(402) = 3.02, *p* = .003 and *t*(402) = 3.87, *p* < .001, respectively). No other effects reached statistical significance.

#### Eye tracking outcomes dependent on the gender attribution

For the incongruent stimuli we were interested whether participants who attributed gender in line with the depicted genital differed from those participants who did not, e.g. attributing male gender when a penis was shown were versus attributing female gender for the same stimulus. We found a significant two-way interaction of stimulus genital and attributions in line with the genital (see Table 5), indicating differences in the accordance of genital and gender attribution between the two incongruent stimuli.

**Fig 4:**
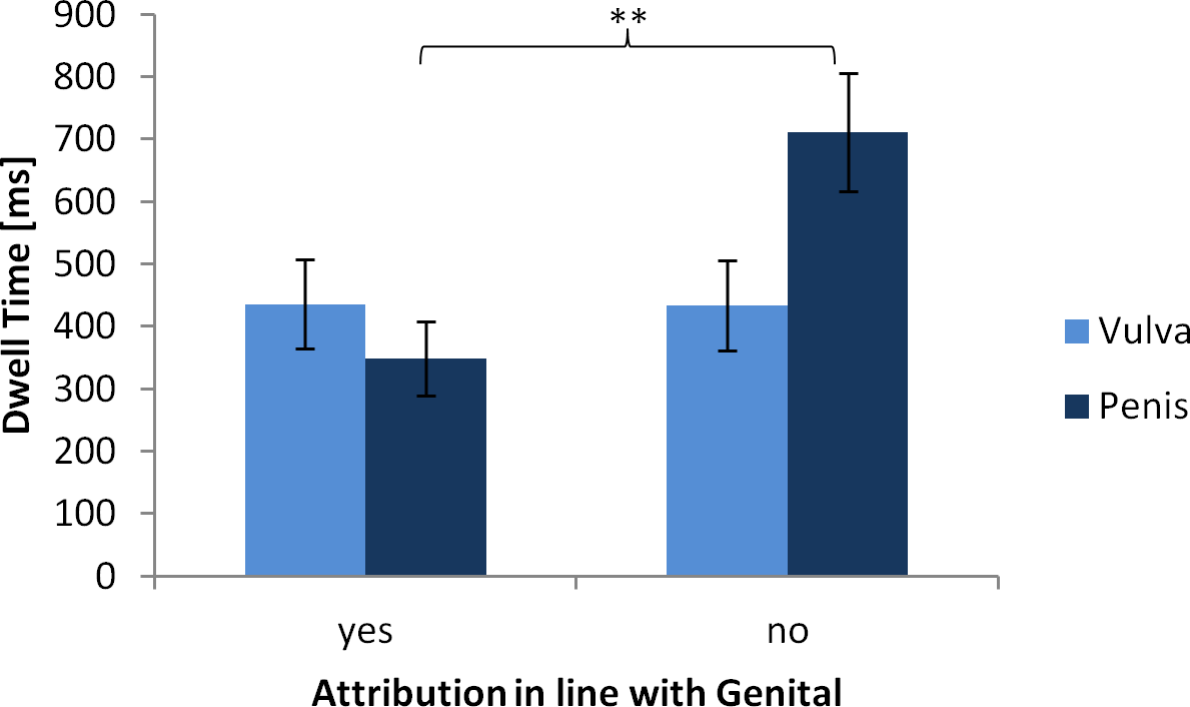
Means and SDs of dwell times [ms] as a function of stimulus genital and attribution in line with the genital. ** p < .01.

Whether a decision was made in line with the vulva did not affect how long participants looked at the stimulus (*p* = .983, see Fig 4). If a penis was present and the gender attribution was made in line with the genital, dwell time did not differ compared to the stimulus with the vulva. But if female gender was attributed to the stimulus with a penis dwell time was significantly greater, (*t*(194) = 4.47, *p* = .001; see Table 6). No other effects reached statistical significance.

**Table 6:** Dwell time [ms] by attribution and genital.

Congruence		Accordance of Attribution^a^	
		M (SE)	95% CI
**Congruent**			
	Vulva	433.78 (72.01)	[291.77, 575.79]
	Penis	711.14 (94.26)	[525.23, 897.04]
**Incongruent**			
	Vulva	435.82 (71.85)	[294.12, 577.52]
	Penis	348.85 (59.04)	[232.42, 465.29]

*Note*. CI = confidence interval.

^a^ Gender attribution made in line or not in line with the depicted genital.

For entry time, the analysis revealed shorter times to first fixate the stimulus for participants who subsequently attributed gender in line with the genital (*M* = 467.92, *SD* = 32.87) than for those who did not (*M* = 627.14, *SD* = 41.80, *t*(161) = 2.99, *p* = .003).

For all other outcome measures, we found no significant effects of stimulus congruence, stimulus genital or subsequent gender attribution.

## Discussion

With the present study we aimed to investigate whether the supposed dominance of the penis in gender attribution, as described by Kessler and McKenna, could still be replicated almost three decades later and to investigate how analyzing eye movements could help understand the attribution process.

### Gender attribution and subjective confidence

Kessler and McKenna understood gender attribution as genital attribution. They ultimately claimed that genital attribution was penis attribution (p.153). Using their original study design, our results support this notion, although the effect we found is less pronounced. Overall, most gender attributions were made in accordance with the genital. When the depicted genital did not reflect the common association of the rest of the presented gender cues (incongruent condition), the attributions were more often in line with the penis than with the vulva. In other words, if a penis was present more male attributions were made than female attributions were made with a vulva present. Nevertheless, instead of the previous almost complete agreement of penis and male gender attribution, 25% of our participants ignored the “reality of the penis” for the incongruent stimulus and attributed female gender. Subjective confidence was higher for the congruent stimuli but did not differ anymore between stimuli with a penis or a vulva within congruence condition.

Besides the relative importance of the actual depicted penis Kessler and McKenna critically stressed the fact that there was a general tendency to think and guess “male”: “Had our participants been asked to attribute gender to an inkblot, they might have responded ‘male’ more often than ‘female’.”([8], pp. 149–150). Indeed, over the past decades evidence of such a “male bias” [44] has been presented for different stimulus types like faces [45–47] or bodies [48, 49], even across cultures [50]. Some researchers actually propose a dual-criterion shift, meaning that participants apply a conservative criterion for judging cues as signaling female while using a more liberal criterion for judging cues as male [51]. However, the male bias is supposed to be especially strong for ambiguous stimuli [45, 48], when inspection time is short [47, 51] or when the male bias is adaptive, e.g. avoiding an aggressive man instead of approaching him [49]. None of those bias-enhancing factors affected the present study. Accordingly, we think that a general male bias might only explain some variance in the response patterns. From a social constructionist perspective the tendency to attribute male gender even when a vulva is present and the penis is not may also have to do with culturally formed representations of the vagina, e.g. considering it as inferior to the penis or even as absent [52]. Also, inequality between men and women, with men being more powerful, still persists today [22, 53], which, as argued by Kessler and McKenna, might promote the tendency to attribute male gender.

### Eye tracking

Applying eye tracking allowed us to analyze how specific stimuli influence viewing behavior in more detail. First, we assessed how long participants looked at each previously designed AOI. Here it became obvious that the regions central for gender attribution were head, chest and genitals. The WHR found to be important for gender attribution previously [29, 30], was not looked at much by our participants. We consider it most likely that the presence of the genital and chest are much stronger cues for gender attribution than the WHR is. Asked how they progressed in attributing gender, most of our participants also stated that they used either the genitals and together with “secondary gender cues”, usually referring to the breast. The lack of visual attention paid to the waist-hip-region could also be due to the simplicity of the stimulus material and possible parafoveal processing of the information [54]. Future research masking stimulus information until a fixation to that region could test this explanation and demonstrate clearly which cues are actively searched for [55].

Eye tracking further revealed that the head AOI is looked at relatively longer than the genital or chest AOI for congruent stimuli. This is in line with previous research suggesting the importance of the face in gender attribution in everyday life and concordantly in the absence of genital cues [39–41]. Though, for the incongruent condition the amount of time spent at each of the three analyzed AOI changed. The head AOI was looked at for the same amount of time while dwell time on the chest and the genital increased. In accordance with previous research we reason that the gender attribution is more difficult for the rather ambiguous incongruent stimuli, leading to overall slower reaction times [56]. The lower subjective confidence ratings, as found for the incongruent stimuli, further support this explanation as lower confidence has been linked to slower reactions [56].

We were surprised not to find any differences with regard to the presented genital concerning both subjective confidence and all eye tracking measures with regard to the genital AOI. If the presence of a penis is in fact a “powerful enough cue to elicit a gender attribution” [8], p.151, we would have expected different results, e.g. shorter dwell times on the penis. Yet, we cannot rule out that participants avoided looking at the ‘private parts’ due to social desirability, an effect that could have been increased by the social presence of eye tracking throughout the experiment [57]. Further research is needed to investigate this option.

We did, nevertheless, find one particularity concerning the interaction effect of stimulus genital and gender attribution on dwell time. If an attribution was made in accordance with the depicted genital, there were no differences between groups. But, when attributing the gender contrary to the genital, the penis made a difference to the total dwell time while the vulva did not. Seeing a penis and nonetheless deciding to attribute female gender seems to need extra cognitive effort, as demonstrated by longer total dwell times [58]. The vulva, on the contrary, is generally considered as less visible and even referred to as absent, which might facilitate ignoring it [52]. Notably, the decision to ignore the presence of the vulva was made by 45% of the participants, while the penis was only ignored by 25%, which further supports this idea.

Since entry times to the stimulus were shorter for participants who subsequently attributed gender in line with the genital, e.g. male gender with a penis present, we speculate that at least some participants had a clearer objective of what they were looking for in the stimulus. However, this idea needs further examination.

### Limitations and future research

As pointed out in the introduction our aim was to replicate the original overlay study in the current social context, which lead us to pose the binary forced choice question for gender attribution. Even though gender is still predominately constructed as either “male” or “female”, the existence of various genders has been widely acknowledged as evidenced by queer characters in mainstream media [15]. While the overall “natural” attitude towards gender might have changed [59], we suggest, that at least for some of the participants investigated, the understandings may have broadened. Some participants’ spontaneous self-reported reasoning indicated awareness of more than two genders. This included considering the incongruent stimulus with a penis as a transgender person in transition and assuming that the last step of the transition would be the sex reassignment surgery, accordingly attributing female gender. Others, however, could think of circumstances for “men without a penis” (e.g. an accident), but not for “women with a penis’, therefore leading to the attribution of male gender to the incongruent stimulus with a penis. As this example suggest, the rationale behind each attribution may vary widely. Forcing participants to attribute male or female gender renders these differences invisible. Although other research has found infrequent uses of additional categories [60], we are wondering how presenting stimulus material with visible genitals is treated differently when gender cues presumably do not match. Presenting various gender categories or open text boxes for the attribution decision could help to find out if some of the male bias might in fact be due to insufficient choices. Qualitative studies could help to understand in more depth the complexity of reasoning before yielding a decision on gender.

Since we decided to replicate the original overlay study and therefore used the original stimulus set, we had to accept the potential shortcomings of the old material. Most importantly, although the face was held constant and intended to be neutral, it was not always perceived as being neutral, but rather as male. Apart from the general male bias previously discussed, this might have had to do with the schematic line drawing. Some participants even saw the upper lip as a moustache while others interpreted the female breast in a male context as a well-trained male chest. However, since the misinterpretation of a cue sometimes happened for female cues in a male context but were never reported in the reverse direction, we argue that the systematic differences in gender attribution are not only due to poorly drawn stimuli because “[the] same ‘female’ cues were perceived as female in a predominantly female context.” ([8], p.150). Also, due to the possible isolation of the specific gender cues, we can precisely attribute the effects found to changes in those cues. Further, the stimulus material included body hair according to the social norms of the 1970s, which could be considered as unusual in the current social context, where partial or complete body hair removal, especially of pubic hair, is the norm for women and becoming increasingly common for men as well [61–62]. Body hair has not only been linked to decreased sexual attractiveness and nonconformity to social norms [61–64] but even to disgust [65], which both have been demonstrated to affect eye movements [28, 66]. Research with more complex or realistic stimulus material (e.g. adding skin) and various combinations of body hair could help to increase the value of eye tracking analysis during gender attribution. This also offers the possibility to identify interactions of observer gender and stimulus gender as has previously been demonstrated for faces [40].

Unlike in the original study, we showed several stimuli to each participant, which might have influenced rating behavior. Some participants stated changing their response pattern from indicating their “overall impression” to merely relying on the genital for gender attribution over the time course. Concerning differences between the congruent and incongruent stimuli the presentation order might have influenced the overall effect, because participants might have realized that the face does not contain any relevant gender information in our stimulus set. Notwithstanding, this does not explain the overall increase in dwell time for the incongruent stimuli. All our participants were raised in Germany and had the same cultural, predominantly binary, understanding of what is natural about gender, which “is itself a social and cultural formulation, discursively produced in an infinite variety of settings” ([1], p.180). As other cultures have traditionally embraced non-binary understandings of gender [67], a comparison with participants socialized differently than ours might reveal interesting differences. The same is true for different age groups, as the male bias has been demonstrated as higher for younger observers [47]. Since both, culture [68, 69] and age [30] affect viewing behavior, applying eye tracking should be considered.

As opposed to other social scientific studies, we had a relatively small sample size due to the effort of eye tracking. Additionally, some of our participants, although without prior knowledge of the overlay study, could be described as particularly gender-aware due to their professional career in medicine or psychology. We would not be surprised if a participant group more naïve to the complexity of gender produced answers more strongly supporting the dominance of the penis. Future research investigating larger samples could reveal whether our results are due to general changes in the natural attitude towards gender or rather are specific for our participant group. If so, it would be interesting to explore how the personal involvement with questions surrounding the sex/gender debate leads to individual variations in gender attribution pattern. In any case, future research using think-aloud-technique or qualitative interviews could unveil variation in reasoning behind individual decisions and help understand possible changes in the natural attitude on a different level.

### Conclusion

More than 30 years after Kessler and McKenna’s foundational work, we demonstrated that gender attribution in binary forced-choice tasks is still predominately genital attribution. Moreover, we presented evidence that the penis persists as a special cue in the decision process and is harder to ignore than the vulva. We interpreted this finding as an increased difficulty with ignoring the penis. We have put this finding into context of the male bias generally found for human perception and the prevailing male dominance in society. We consider it hence appropriate to conclude by once more citing Kessler and McKenna: “The power of the penis lies not in its absence […], but in its presence” (p.153).

## Supporting information

S1 Dataset Original participant and behavioral data. (CSV)
